# Electronic polymers and soft-matter-like broken symmetries in underdoped cuprates

**DOI:** 10.1038/ncomms8691

**Published:** 2015-07-06

**Authors:** M. Capati, S. Caprara, C. Di Castro, M. Grilli, G. Seibold, J. Lorenzana

**Affiliations:** 1 Dipartimento di Fisica, Università di Roma Sapienza, Piazzale Aldo Moro 5, I-00185 Roma, Italy; 2 ISC-CNR, Via dei Taurini 19, I-00185 Roma, Italy; 3 CNISM Unità di Roma Sapienza, Piazzale Aldo Moro 5, I-00185 Roma, Italy; 4 Institut für Physik, BTU Cottbus—Senftenberg, PO Box 101344, 03013 Cottbus, Germany

## Abstract

Empirical evidence in heavy fermion, pnictide and other systems suggests that unconventional superconductivity appears associated to some form of real-space electronic order. For the cuprates, despite several proposals, the emergence of order in the phase diagram between the commensurate antiferromagnetic state and the superconducting state is not well understood. Here we show that in this regime doped holes assemble in ‘electronic polymers'. Within a Monte Carlo study, we find that in clean systems by lowering the temperature the polymer melt condenses first in a smectic state and then in a Wigner crystal both with the addition of inversion symmetry breaking. Disorder blurs the positional order leaving a robust inversion symmetry breaking and a nematic order, accompanied by vector chiral spin order and with the persistence of a thermodynamic transition. Such electronic phases, whose properties are reminiscent of soft-matter physics, produce charge and spin responses in good accord with experiments.

## Abstract (web summary)

High-Tc superconductivity is thought to be associated with spatial electronic ordering, which for cuprates is not well understood yet. Here the authors use Monte Carlo simulations to show the emergence of a soft-matter-like electronic phase between the antiferromagnetic and the superconducting states.

## Graphical abstract

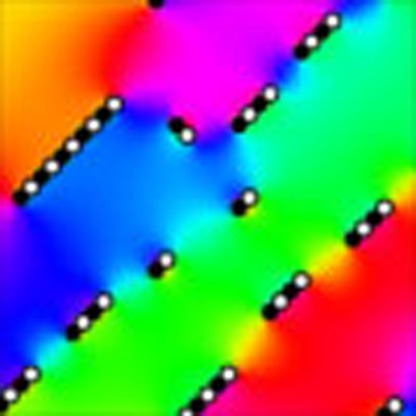

The anomalous behaviour of several physical quantities in cuprates above the superconducting transition temperature suggests that high-temperature superconductivity emerges close to a quantum critical point between a broken-symmetry state and the disordered phase, in analogy with heavy fermion materials, pnictides and organics^1^. However, identifying the broken-symmetry phases has proven much more difficult than in other materials, despite several ‘gold rushes' in the underdoped region of the phase diagram, triggered by the observation of stripe order^2^,^3^, nematic order^4^,^5^,^6^,^7^, time-reversal symmetry breaking^8^ and incommensurate charge-density-wave order^9^.

While some form of charge order (CO) is well established^10^, an important question is how this order is formed starting from the two extremes of the phase diagram. When coming from the high-doping region, CO seemingly arises as a second-order instability of the uniform strongly correlated metallic state, producing incommensurate charge density waves driven by magnetic^11^,^12^, phononic^13^,^14^ or mixed^15^ microscopic mechanisms. On the other hand, the occurrence of CO from the Mott insulating low-doping side is more directly tied to the tendency of Mott antiferromagnets to expel and segregate charges^16^,^17^,^18^,^19^. This latter region is the playground of our present work.

Recently, it was pointed out^20^,^21^ that, at very low doping, charge segregation may acquire features related to the occurrence of topological excitations in doped antiferromagnets. The starting point is the observation that holes in an antiferromagnet induce a vortex (V) or antivortex (A) texture in the surrounding spin ordering. While isolated vortices are energetically expensive, a VA pair is stable^22^,^23^,^24^,^25^, because its annihilation is hindered by the strongly correlated character of the doped holes and the disturbance of the antiferromagnetic background rapidly dies out at large distances. As in early proposals^26^,^27^ inspired by the work of Villain^28^,^29^, these VA ‘dimers' explain the extremely rapid destruction of long-range antiferromagnetic order with doping.

In the present scenario, the dimers or ‘nematogens' self-organize and give rise to ‘electronic soft-matter' effects^30^. Specifically, the dimers may undergo a ‘polymerization process', triggering charge segregation into segments, tightly bound to V and A spin textures. These segments not only align forming a nematic state, but can also break inversion symmetry^28^,^29^ owing to their intrinsic topological dipolar character (associated with the V and A at the end points of the ‘polymer'). This state, which was named ferronematic^20^,^21^, is accompanied by a spin spiral state sustaining a net spin current. At large scales, this feature is reminiscent of other proposals^31^,^32^,^33^,^34^, which are however based on impurity states instead of the polymer states that are central to our results. A ferronematic phase was proposed also to occur in ultra-cold dipolar Fermi gases of atoms^35^.

We pose here the following fundamental questions: which other phases can be sustained by the electronic polymers, how are they affected by quenched disorder, what is the fate of the thermodynamic phase transitions expected in ideally clean systems, and how their characteristic temperature scales emerge from the (usually much higher) electronic scales of the system. In order to study the problem at the large length scales probed by experiments, we carry out a multiscaling approach starting from a microscopic model and derive a mesoscale effective model treated with Monte Carlo. We obtain a rich phase diagram for the electronic polymers as a function of temperature and disorder, which allows to rationalize the charge and spin responses observed experimentally.

## Results

### Numerical simulations

We start from the very low-doped regime of few holes in the spin antiferromagnetic background of a CuO plane modelled by a one-band Hubbard model. We study the dimers at mean-field level in the Gutzwiller approximation (see Methods section; Supplementary Note 1 and Supplementary Fig. 1). With realistic parameters for La_2−*x*_Sr_*x*_CuO_4_, the most favourable configuration for two holes is along the diagonal of a plaquette with a planar dipolar distortion of the antiferromagnetic background. The latter can be visualized as due to a V and an A centred close to (but not exactly at) the vertices of the plaquette and forming a ‘topological dipole' (TD). There is another two-hole mean-field solution that is non-planar and consists of a skyrmion texture^22^, which, for the present parameters, is ∼100 K higher in energy than the TD, and therefore will be neglected at low temperatures in the following.

Studying metastable planar configurations in which two or more of these TDs are arranged with different positions and orientations (Supplementary Figs 2 and 3 and Supplementary Note 1) we find, as expected, that at large distances holes interact through a logarithmic interaction^36^ between their topological charges, whereas at short distances their interaction is modified by quantum effects related to the overlap of the hole wave functions. The logarithmic interaction stems from the fact that for planar textures, the long-range behaviour can be captured by an XY model^26^,^27^. Notice that we are not claiming that the symmetry of the model is reduced from Heisenberg to XY. Indeed each texture has a zero mode related to the change of the plane that contains the spins, as it should for an O(3) symmetric model. However, contrary to what would happen for a single V in the pure Heisenberg model, the textures have no unstable modes^21^ that would break the planar character of the texture, that is, they are locally stable and therefore their energy is correctly captured by a planar magnetic model.

In order to enable simulations in large systems, we do not consider explicitly the spin degrees of freedom but integrate them out to generate effective interactions among topological charges. While this is an enormous computational advantage, it limits our simulations to low doping (*n*_h_
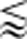
0.1 holes per unit cell) where spin currents are small on average and the superposition principle is valid, allowing for a mapping of topological charges onto a two-dimensional (2D) Coulomb gas^36^. The effective interaction among topological defects, needed for the Coulomb gas model, is obtained by fitting the energy of several metastable zero-temperature Gutzwiller approximation solutions obtained in the Hubbard model.

**Figure 1:**
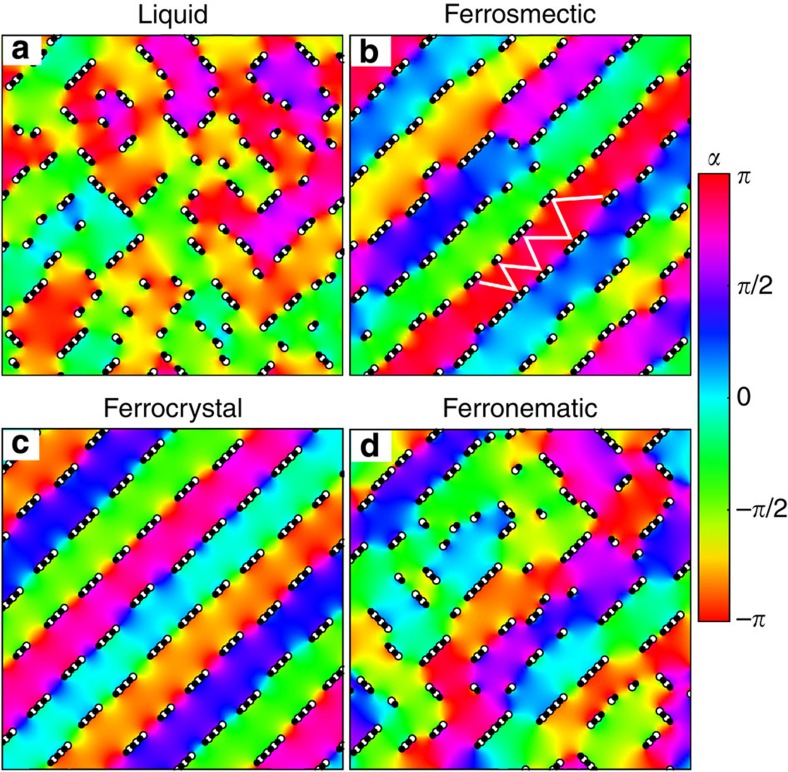
Charge and spin configurations in the different phases. White and black circles represent the positive and negative topological charges, respectively. The different colours denote the angle-*α* of the staggered magnetization. The images are Monte Carlo snapshots in the absence of quenched disorder (**a**–**c**) in the thermally disordered phase with *T*=50 K (**a**), in the ferrosmectic phase at *T*=38 K (**b**), in the ferrocrystal phase at *T*=8 K (**c**) and in the ferronematic phase at *T*=40 K (**d**), which appears in the presence of quenched disorder (*Q*_ion_/*Q*_rep_=0.125). The white lines in **b** highlight the ‘triangular' arrangement of the segments.

As a consequence of the interaction mediated by the antiferromagnetic background, when a large even number of holes is added to the system, these tend to bind into a single polymeric chain of alternating topological charges, ending with a V and an A. Adding the real three-dimensional (3D) long-range Coulomb repulsion among holes, whose strength is measured by a parameter *Q*_rep_ (see Methods section), these long polymers break into smaller polymers, as shown in Fig. 1 and Supplementary Fig. 5.

We work at temperatures *T* smaller than the binding energy of individual VA pairs (≈100 K), so that the number of unbound topological charges is negligible. Therefore, our basic constituents in the Monte Carlo computations are the TDs. These are modelled by a bound V and A, each moving on the sites of a square lattice, with the topological charge adjusted so that the dipole moment matches the Gutzwiller computations (see Methods section). Since there are no topological constraints on the charge ±*k* of the V and A, they turn out to be fractional, *k*≈0.8 (Supplementary Note 1).

A crucial problem in cuprates is to determine how disorder affects the ordered phases of the ideal ‘clean' system^7^,^37^. In order to address this issue, the holes attached to the topological charges are subject to a ionic disorder potential with strength *Q*_ion_, generated by the counterions out of the CuO_2_ plane (see Methods section, Supplementary Note 2 and Supplementary Fig. 4). The magnitude of *Q*_ion_ is difficult to estimate because it depends on screening processes not comprised in the model. Therefore, we treat *Q*_ion_/*Q*_rep_ as a phenomenological dimensionless parameter that characterizes the amount of disorder.

We consider a *L* × *L* square cluster with even number of holes *N*_h_, corresponding to *N*_h_/2 TDs. Although we explored various fillings, for the sake of definiteness in the present communication, we report the results for the typical case *L*=100 and *N*_h_=300, corresponding to a hole doping *n*_h_=0.03.

To characterize the broken symmetries, we define a nematic order parameter *φ*(*T*) (equation (5) in Methods section), which becomes different from zero when the *C*_4_ rotational symmetry of the lattice is broken. We also define the polarization of the system as the normalized sum of all the TD moments projected on the (1,1) and (1,−1) preferred directions (cf. Methods section, equations (6) and (7)). A nonzero polarization in the system implies a breaking of inversion symmetry of the magnetic texture. Vector chiral spin order is characterized by the chirality 
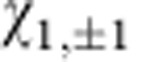
 (see equation (8) in Methods section). Finally, the charge and spin structure factors allow us to further characterize the various phases.

The Monte Carlo computations find at high temperature a classical liquid of dimers that tend to form longer polymers as temperature is lowered (Supplementary Fig. 6), and to align along the diagonal directions, which are energetically favourable. Figure 1a reports a snapshot of this high-temperature phase taken during the Monte Carlo evolution.

### Clean system

**Figure 2:**
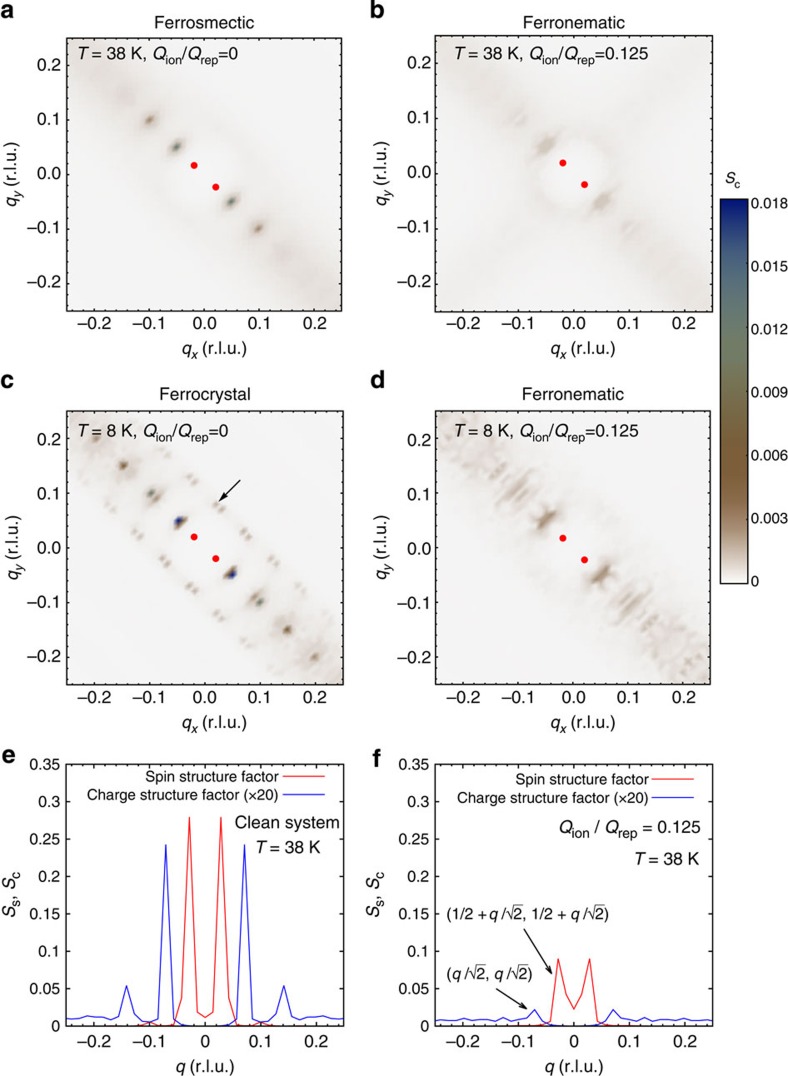
Charge and spin structure factor. Density plots in the 2D reciprocal space for the charge structure factor for the clean system at (**a**) 38 K and (**c**) 8 K, and the charge structure factor for the system with *Q*_ion_/*Q*_rep_=0.125 at (**b**) 38 K and (**d**) 8 K. The red solid circles represent the position of the spin peaks in the reciprocal space (shifted by *q*_AF_). The arrow in **c** shows a ferrocrystal peak. We also show the diagonal cut of the charge and staggered spin structure factor in the 2D reciprocal space at 38 K for (**e**) the clean system and (**f**) the system with *Q*_ion_/*Q*_rep_=0.125. In order to see more clearly the effects of the broken *C*_4_ symmetry, the averages are restricted to configurations with *φ*≥0 corresponding to the expected response in a single-domain sample.

For the clean system (*Q*_ion_=0) we find that when *T* is low enough, the segments orient to form a state with *C*_4_ symmetry breaking. As is clearly visible in Fig. 1b (see also Supplementary Fig. 5), associating the segments with ‘polymers', the low-*T* phase corresponds to the so-called smectic order of soft matter^38^, in which the system has long-range positional order in one direction, with periodicity 
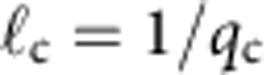
 (*q*_c_ being the magnitude of a characteristic wave vector in reciprocal lattice units (r.l.u.)), but remains ‘liquid' in the other direction. This manifests as sharp (resolution limited) peaks in the structure factor along the diagonal of the Brillouin zone (Fig. 2a), that is, perpendicular to the preferred polymer direction, signalling long-range positional order. As is shown in Fig. 2e, the main spin peak is 20 times higher than the charge peak, because the spectral weight of the latter is spread over a wider range of wave vectors, owing to the highly anharmonic charge distribution. (Notice that both structure factors, as defined in Methods section, have the same normalization.) Regarding CO, this state has the same symmetry as a diagonal stripe state. However, the charge is uniform along the stripe direction only after thermal fluctuations have been taken into account. In addition, this state breaks inversion symmetry, that is, TDs tend to point in the same direction, thus we call it ferrosmectic.

The ferro ordering associated with this and other phases is not trivial. Indeed, in contrast to dipoles on a cubic lattice in three dimensions, it would not occur if, for example, the TDs were arranged at fixed positions on a square lattice. This stems from the 2D dipole–dipole interaction, which is ferroelectric in a nearly head-to-tail configuration of the dipoles, but is antiferroelectric for a side-by-side configuration. In our model, the ferro tendency wins because the real Coulomb interaction between the electrically charged holes favours short-range triangular arrangements of the segments, that is, segments in one row tend to face gaps in the neighbouring rows as is clear in Fig. 1b (highlighted by white lines) and Fig. 1c, so that the side-by-side arrangements are rare. The colours in Fig. 1 show the phase of the local staggered magnetization. In Fig. 1b–d, the phase increases monotonically along one diagonal, indicating that these phases have long-range vector chiral order, that is, *χ*_1,−1_≠0 or *χ*_1,1_≠0.

Upon further lowering the temperature, the ferrosmectic phase keeps the ferro ordering (and the vector spin chirality), but forms a Wigner crystal for *T*
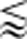
10 K as shown in Fig. 1c. This ‘ferrocrystal' manifests as additional resolution limited off-diagonal peaks in the charge structure factor (as the one indicated with an arrow in Fig. 2c) and which again signal long-range charge and spin order (within our system size).

### Effect of disorder

The properties of the phases change markedly upon the introduction of quenched disorder. The ferrosmectic and ferrocrystal peaks broaden and weaken very rapidly (Fig. 2b,d,f), thus long-range positional order is lost and the ferrosmectic–ferrocrystal transition is smeared. Remarkably long-range nematic and vector chiral order (accompanied by inversion symmetry breaking) remain at finite disorder, and the phase becomes the ferronematic state proposed in refs 20, 21. Figure 2f shows how CO is almost entirely destroyed by small disorder. Long-range spin order also is destroyed but short-range spin order, signalled by incommensurate peaks, whose width is well resolved in our system size, persists. For *Q*_ion_/*Q*_rep_>0.25 even the broad incommensurate magnetic peaks disappear (Supplementary Note 4 and Supplementary Figs 6–8). This would contradict experiments, we thus estimate *Q*_ion_/*Q*_rep_<0.25 in real systems.

The red solid circles in Fig. 2a–d show the vectors 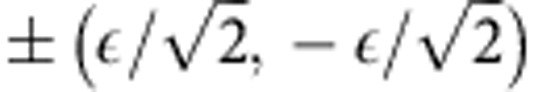 r.l.u., where 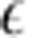 is the magnetic incommensurability, that is, for the orientation of Fig. 1b,c magnetic peaks appear at 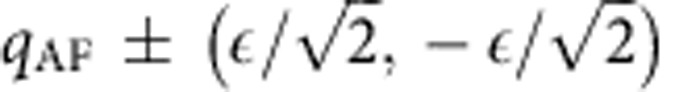 r.l.u., with *q*_AF_=(0.5,0.5) r.l.u. From all panels we see that the main magnetic peaks appear at half the incommensurate wave vector of the main charge peaks. At first sight, this relation, well known for spin collinear stripes^39^,^40^, is surprising here, since the incommensurability should be linked to the topological polarization^20^. However, close inspection of Fig. 1b reveals that each segment acts as an antiphase domain wall for the antiferromagnetic background, yielding jumps of the phase of the magnetic order parameter close to *π* upon crossing the line of polymers. On the other hand, the phase is approximately constant in between two polymer rows. Thus, the magnetization behaves similarly to the case of a collinear stripe array. Spin canting produces small corrections to the ‘factor of two' relation, which are below our momentum resolution to be visible in Fig. 2.

**Figure 3:**
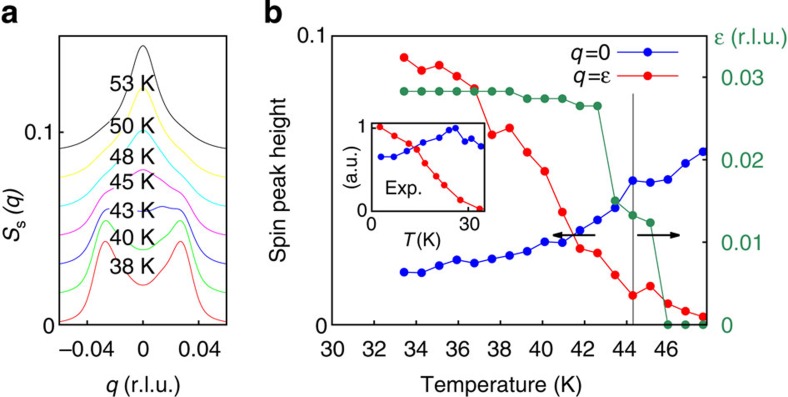
Commensurate–incommensurate transition (**a**) Diagonal cuts of the spin structure factor for different temperatures as a function of momentum with *q* defined as in Fig. 2f and disorder *Q*_ion_/*Q*_rep_=0.125. The peaks have been convoluted with a Gaussian (s.d. 0.041 r.l.u.) to take into account a finite experimental resolution. (**b**) Height of the structure factor shown in **a** at the commensurate antiferromagnetic wave vector (blue) and at the incommensurate position with respect to the background (red) as a function of temperature. The green data (right scale) show the incommensurability as a function of temperature. The vertical line marks the ferronematic transition. The arrows help to identify the scale associated with the data. The inset shows the experimental peaks height from ref. 60 for doping *n*_h_=0.0192, which is slightly below the complete disappearance of static antiferromagnetic order as revealed by muons. The evolution of the incommensurate peaks has been shown to be continuous^41^ across the critical doping *n*_h_=0.02.

Raising the temperature at small disorder, the broadened spin and charge peaks gradually decrease without any sign of a sharp transition in the intensity (Fig. 3 and Supplementary Fig. 6, respectively) as also observed experimentally at similar dopings (see inset of Fig. 3b and ref. 41). In contrast, studying the polarization and nematic order parameter distribution, we find that the transition from the ferronematic to the melted polymers is of first order and remains sharp for our system size (Supplementary Note 5). Thus, a thermodynamic transition persists even in the presence of disorder. The thermodynamic transition temperature is signalled by a change of behaviour in the magnetic structure factor from commensurate to incommensurate, providing a simple experimental tool to detect the transition line (Fig. 3a). This is because the incommensurability 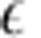 is related to the degree of polarization in the system and thus acts as an order parameter^20^.

### Phase diagram

**Figure 4:**
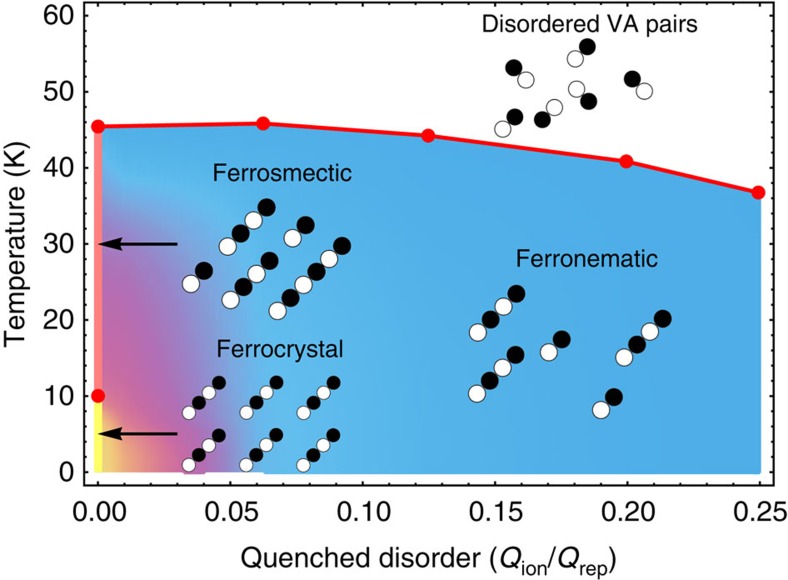
Phase diagram as a function of temperature and disorder strength. The yellow (pink) thick line at zero disorder corresponds to ferrocrystal (ferrosmectic) long-range order. The yellow region is short-range ferrocrystal order, whereas the magenta region corresponds to the short-range ferrosmectic order. At finite disorder, below the red line, the system has long-range ferronematic order (light blue region) while a polymeric liquid is found above the red line, up to the highest temperatures reached in our study.

Figure 4 reports the phase diagram obtained from the above analysis. The ferrocrystal (thick yellow line) and ferrosmectic (thick pink line) phases are well defined only in the absence of disorder. At finite disorder, they survive as short-range-ordered states. This is indicated by the yellow shade for the ferrocrystal and by the magenta shade for the ferrosmectic state. The light blue region is the long-range-ordered ferronematic state, whereas the red line indicates the first-order transition to a liquid of short polymers. We never find a purely nematic phase, characterized by a nonzero nematic order parameter but zero polarization and zero global vector chiral spin order. The last phase, which is allowed by our model, could possibly be stabilized in a different parameter regime, as an intermediate phase between the ferronematic and the disordered phase.

Our results are in good qualitative agreement with the phase diagram obtained by completely different methods in ref. 37. On the other hand, we find an additional inversion symmetry breaking, and we provide realistic estimates of the parameters of the model, of the experimentally measurable structure factors and of the characteristic temperatures of the transitions.

The order of magnitude of the transition temperature to a polarized state can be estimated using a mean-field approximation. For dipoles in two dimensions at random positions but with a nonzero average dipole moment 〈*p*〉, the dipolar field can be computed using elementary electrostatics to be *E*_d_=2*π*^2^*ρ*_s_〈*p*〉*n*_h_/*N*_c_, where *ρ*_s_ is the magnetic stiffness of the system and *N*_c_ is the number of charges per segment. Assuming a mean-field approximation where the dipoles, of strength *p*_0_≡
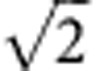
(*N*_c_−1)*k*, can fluctuate in four possible orientations (↑, →, ↓ and ←) we obtain the ordering temperature,


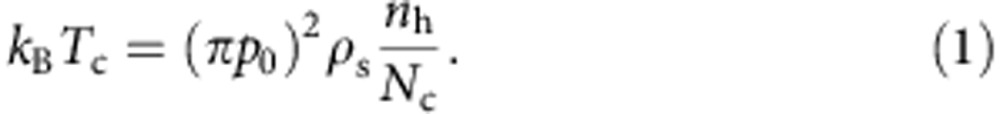


With the present parameters and assuming *N*_c_≈4 (Supplementary Fig. 6), we find *T*_c_≈196 K. This is of the correct order of magnitude (that is, much smaller than the original electronic scales), taking into account that we have neglected the positional entropy that will reduce *T*_c_.

Both ferrosmectic and ferrocrystal charge orderings are not commensurate. Thus, they break a continuous [U(1)] symmetry in two dimensions. Even in the presence of infinitesimal disorder strict long-range order is forbidden^42^ and one finds quasi long- or short-range order. In contrast, the nematic order parameter breaks a discrete (Z_2_) symmetry and is much more robust against disorder. In our computations, we have in addition vector chiral spin order (or equivalently a topological polarization) that also breaks a discrete (Z_2_) symmetry, but which does not couple linearly to the local disorder, in contrast to the nematic order parameter^43^,^44^. General arguments indicate that the discrete symmetry breaking should be much more robust than the breaking of a continuous symmetry^37^,^45^, as we indeed find. We expect the nematic order to behave similarly to the random field Ising model: lacking long-range order in a strictly 2D system, but ordered within a correlation length, which can be exponentially large for small disorder^46^, favouring a crossover to 3D long-range order in the presence of a small inter-layer coupling^44^,^47^.

Since the ferronematic state has short-range spin order and long-range vector chiral order (at *q*=0 wave vector), it can be identified with the chiral spin liquid believed to take place in frustrated magnets^48^,^49^,^50^.

## Discussion

Our results allow us to rationalize several experimental findings, and imply some predictions that have not yet been tested.

Experiments show that hole doping destroys commensurate antiferromagnetic order much more rapidly than what would be expected by site dilution^51^,^52^. Figure 2f shows that this is explained by a small density of TDs. The ability of VA pairs to rapidly depress commensurate ordering was noticed before^26^,^27^, although these authors did not consider the collective ordering of the dipoles.

Incommensurate spin scattering has been detected in the early days of high *T*_c_ (ref. 53) and interpreted in terms of stripes^2^,^3^. However, stripes are associated with charge modulations that are extremely hard to measure, in contrast to spin modulations. CO generally emerges associated with a structural distortion close to *n*_h_=1/8, which can be controlled by codoping with Nd^2^,^3^ or doping/codoping with Ba^54^,^55^,^56^. All these observations of CO are at doping close to *n*_h_=1/8. The intensity of CO decreases strongly with underdoping and extrapolates to zero around *n*_h_≈0.09 (ref. 56). To the best of our knowledge, incommensurate static CO has never been reported in the present, heavily underdoped regime, in contrast to incommensurate spin order^57^, which persists. This dichotomy is explained by our simulations that, while reproducing the incommensurate spin ordering, show very weak charge-ordering peaks, barely emerging from the background noise, even for weak disorder (Fig. 2f).

Close to *n*_h_=1/8 magnetic Bragg peaks appear quite sharp and often resolution limited^3^,^56^,^58^ indicating long-range order. As doping is reduced, static peaks are still observed but become broad with a well-resolved width of the order of the incommensurability, indicating a correlation length of the order of the spin periodicity^56^,^57^. This is in excellent agreement with our magnetic structure factor in Fig. 2f. We interpret this feature as an indirect signature of long-range vector chiral spin order without long-range magnetic order, that is, the ferronematic state we propose. For *n*_h_=0.03, experimental magnetic peaks have been detected with incommensurability 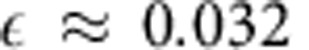 (ref. 41), which is in good agreement with our computations yielding 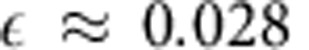 at low temperature.

Neutron-scattering experiments in Y-based materials have shown^4^,^5^ that the magnetic incommensurability as a function of temperature behaves as an order parameter. Such a behaviour is naturally explained by our model, where the incommensurability, in the presence of weak or no CO, is closely linked to the topological polarization^20^, which is an order parameter (Fig. 3). Furthermore, we propose that the temperature at which the static magnetic structure factor changes from a double peak structure to a single peak structure is a proxy of the thermodynamic critical temperature below which long-range chiral spin order is established.

The transition from incommensurate behaviour to commensurate behaviour has been observed also in the specific La family we focus on in the present computations. Indeed, experimental low-energy inelastic neutron-scattering peaks as a function of temperature reported in Fig. 5a of ref. 59 (see also ref. 57) show the same behaviour as we find for the static structure factor. However, the transition from two incommensurate peaks to an antiferromagnetic commensurate peak takes place ∼55–100 K. On the other hand, quasistatic scattering shows a transition at ∼20–30 K (see refs. 41, 60, 61 and inset of Fig. 3b). Our computations provide an energy-integrated structure factor that is expected to show the transition at an intermediate temperature between the inelastic and quasistatic cases. Indeed, we find the commensurate–incommensurate transition at ∼45 K fully consistent with the neutron-scattering measurements. Such an agreement on the temperature scales and qualitative behaviour further supports our identification of the low-temperature state observed in cuprates as a long-range-ordered ferronematic.

Notice that, in contrast with the small ordering scales, we find that the starting point electronic Hamiltonian has bare electronic scales of the order of 3,000 K or more. This strong reduction of energy scales indicates that our multiscale modelling has identified the correct dynamical variables of the problem. Equation (1) shows that the energy scale is set by the magnetic stiffness and the density. Notice also that the proposed thermodynamic transition occurs at a temperature much lower than the pseudogap temperature (≈300 K) that instead nearly extrapolates to the Néel temperature of the undoped sample^62^.

At even lower temperatures of the proposed ferronematic transition, a so-called cluster spin glass state is observed consisting of strongly coupled clusters of spins with weaker coupling among clusters^51^,^52^,^61^,^63^,^64^,^65^. The ferronematic state of Fig. 1d corresponds precisely to this physical picture.

Equation (1) predicts a linear relation between doping and the ordering temperature. However, for *n*_h_>0.02, one should take into account that the finite magnetic correlation length, the expected weakening of the stiffness by doping and the breakdown of the linear regime for the topological charges will lead to a slowing down of the doping dependence. Interestingly, for hole content *n*_h_<0.02, the temperature at which the cluster spin glass state is observed is linear with the doping^63^,^64^,^65^ consistent with our proposal. Numerically, we find that the temperature of the ferronematic transition increases approximately linear with doping as *T*_c_∼1,500 K *n*_h_ compared with the experimental behaviour *T*_c_∼815 K *n*_h_. Our larger slope may be owing to an overestimation of *ρ*_s_, a smaller *N*_c_ and/or dynamical effects that may give an apparent shift of the transition.

In the presence of spin–orbit coupling, long-range vector chiral spin order gives rise to a real electric polarization, that is, the system becomes an improper ferroelectric^66^. Unfortunately, this effect is hard to observe because as soon as the system becomes metallic, it cannot support a finite electric polarization. Notwithstanding, a finite ferroelectric polarization has been reported at low temperatures in oxygen^67^ and Li^68^-doped La_2_CuO_4_, the samples having a strongly insulating character. The fact that the effect appears independently of the dopant, and that the remnant polarization can be oriented along different axes with external fields, clearly points to a magnetic origin of the ferroelectric polarization. Furthermore, more recent experiments show a clear correlation between magnetoelectric effects and stripe orientation in Sr-doped La_2_NiO_4_, suggesting that stripe effects are involved (C. Panagopoulos, personal communication). Experiments at finite frequencies suggest that inversion symmetry breaking sets in at temperatures higher than the temperatures at which the sample is insulating enough to support a static polarization. All these experiments support our conclusion that underdoped cuprates show long-range vector chiral spin order.

A possible test to our model would require second harmonic generation to detect inversion symmetry breaking in non-insulating samples. We predict that in the ferronematic phase, the inversion symmetry breaking should track the behaviour of the incommensurability as a function of temperature. This relation, however, will break down in the collinear stripe phase found ∼*n*_h_=1/8.

With the present method, we cannot access quantitatively the crossover to collinear stripes. In this regime, the mapping to the Coulomb gas breaks down due to nonlinear effects. However, one can anticipate that the average length of the segments will keep growing with doping, leading to a concomitant increase of the ferrosmectic correlation length. According to our findings, the disorder induced by the dopants will partially counteract this increase, but the associated impurity potential will also be progressively screened, opening the possibility that segments coalesce into stripes with long-range order and narrow magnetic peaks.

We thus propose that underdoped cuprates have a long-range broken symmetry state at low doping. This puts the cuprate phase diagram into the same class of phase diagrams of a wide class of materials^1^ in which unconventional superconductivity emerges from a phase characterized by real-space electronic long-range order.

## Methods

### Model

Treating the single-band Hubbard model within a Gutzwiller approximation, a single hole in the antiferromagnetic background is found to form a spin polaron, while two holes tend to occupy the cores of a spin V and A that attract each other, thereby lowering their energy. The long-range part of this texture is treated using generalized elasticity^38^ and exploiting the correspondence between a spin V and a 2D Coulomb charge^36^. In the absence of disorder and holes, the magnetic correlation length is expected to be very large but finite due to thermal fluctuations. This provides a natural cutoff at a distance *λ* for the long-range interactions between topological charges at large distances^26^,^27^. Therefore, the interaction energy is well described by


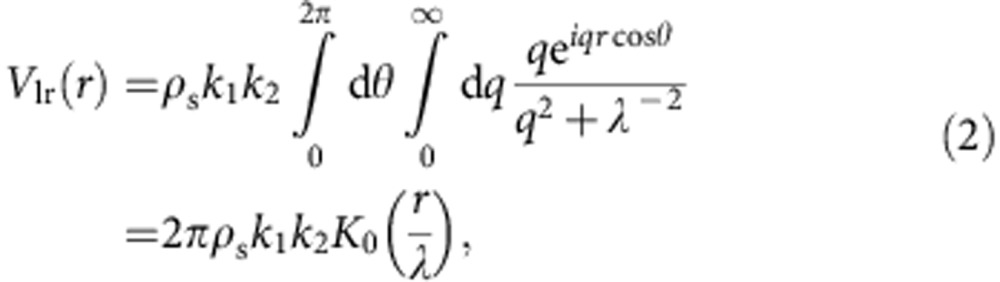


where *k*_1,2_=±*k* are the topological charges (in our case, *k*≈0.8) and *K*_0_ is the zeroth order modified Bessel function, which reproduces the logarithmic interaction at short/intermediate distances (*r*
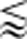
*λ*) and decays exponentially at long distances (*r*≫*λ*). We have checked that changing *λ*, the results are substantially the same, as long as it remains larger than the typical distance between the segments. In the simulations, we take *λ* of the order of the system size for numerical convergence purposes.

While equation (2) reproduces well the energy of Gutzwiller calculations for the single-band Hubbard model at large distances, as expected, it fails at short distances where the short-range physics of the Hubbard model becomes relevant. Therefore, the interaction potential also includes short-range terms extrapolated from the Gutzwiller calculations (Supplementary Note 1).

Furthermore, each topological charge arising from the spin texture corresponds to a positive electrically charged hole in the CuO_2_ planes of the doped cuprate. Therefore, our model includes also the 3D Coulomb repulsion potential which, for two holes at a distance *r*, is parametrized as


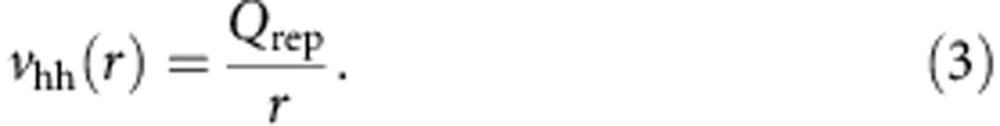


Here *Q*_rep_, incorporating, for example, the static dielectric constant, represents the strength of the repulsion and is another parameter of our model. We fix *Q*_rep_/*a*=49 meV, where *a* is the in-plane lattice constant, so that the average number of holes in a polymer, for very low density, is *N*_c_≈2. As the density increases, this number tends to increase too^20^, yielding the results of Supplementary Fig. 6 for the present density (*n*_h_=0.03).

Finally, the charged holes doped into the CuO_2_ planes leave back negative countercharges. For instance, in La_2−*x*_Sr_*x*_CuO_4_, which we take as a prototype cuprate, negative Sr ions randomly replace La atoms between two consecutive planes. We therefore introduce disorder, generating a random distribution of point-like negative charges, one for each positive hole in the plane, which act as pinning centres for the carriers in the plane (Supplementary Note 2). The ions are located out of plane, at a distance 
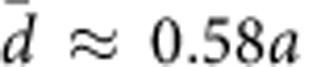
, from the centre of the in-plane plaquette. Each impurity interacts with the holes in the plane through an attractive 3D Coulomb potential


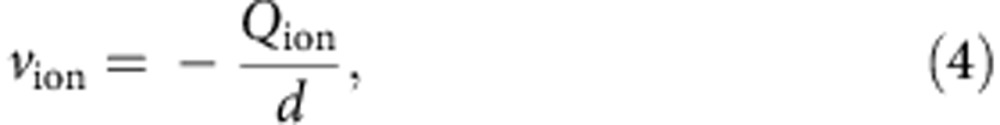


where *d* is the distance between the hole and the impurity, and the strength of the interaction *Q*_ion_ measures the intensity of disorder. This procedure thus produces a disordered potential in the plane in which holes and their associated topological charges move. We show one realization of the impurity potential in Supplementary Fig. 4.

### Characterization of the phases

To characterize *C*_4_ rotation symmetry breaking, we introduce the nematic order parameter


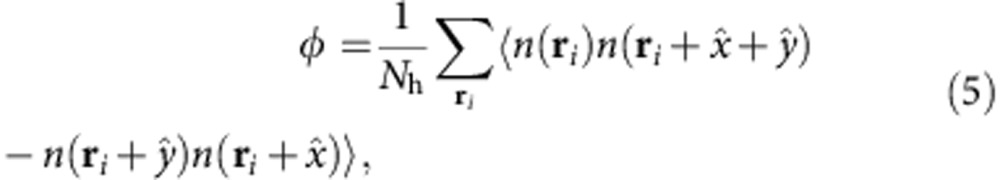


where *n*(**r**_*i*_) is the number of holes on the site labelled by **r**_*i*_, and 
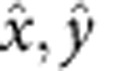
 are unit vectors along the corresponding directions of our 2D square lattice. The angular brackets imply thermal average, *N*_h_ denotes the total number of charges, and the sum runs over all the lattice sites. To characterize inversion symmetry breaking, we introduce the polarization *P*=(*P*_*x*_,*P*_*y*_), as the normalized sum of all the TDs. Since in our model diagonal polarizations are favoured, we introduce the components


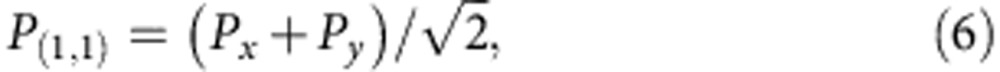



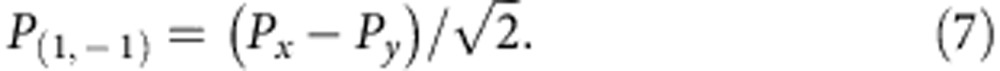


Vector chiral spin order is characterized by the parameter


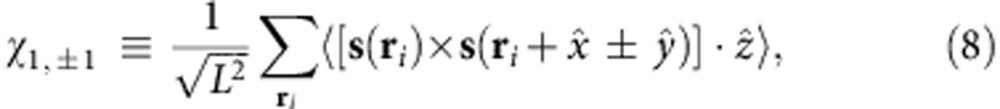


where **s**(**r**_*i*_) is the local spin density.

Our Monte Carlo calculations also yield the thermal averages of the static charge (c) and spin (s) structure factors, *S*_c_(**q**)=(1−*δ*_**q**,0_)|*K*_c_(**q**)|^2^/*L*^2^ and *S*_s_(**q**)=|*K*_s_(**q**)|^2^/*L*^2^, where


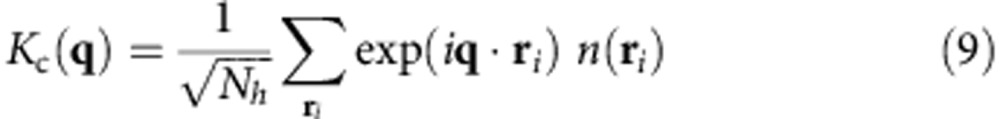



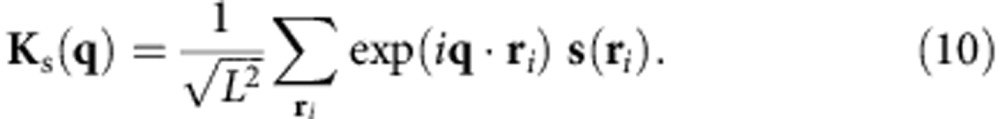


With these definitions and using unit-length spins, the structure factors have the same normalization and satisfy Σ_**q**_*S*_s,c_(**q**)=1−*n*_h_.

The ferronematic–ferrosmectic crossover in Fig. 4 was characterized analysing the height of the main charge peak as a function of temperature.

### Monte Carlo analysis

We carried out Monte Carlo calculations exploiting the parallel tempering technique. To analyse the spin degrees of freedom for a given configuration, we attach to each topological charge the structure of a (anti)vortex in the spin background, and we perform a linear superposition, allowing then each spin to relax according to the XY Hamiltonian^36^. Supplementary Figure 1b reports an example for the case of two VA pairs aggregated in a four site segment. Further examples with a detailed view of the segments of the corresponding relaxed spin structures and of the resulting spin currents are reported in Supplementary Note 3 and Supplementary Fig. 5.

The temperature step of our simulations is 0.4 K (0.8 K) for the clean (disordered) case. To better determine the various phases, at each temperature, we construct a histogram over the Monte Carlo history defined on a 3D grid spanned by the order parameter (*φ*,*P*_(1,1)_,*P*_(1,−1)_) (ref. 69). The probability for a value (*φ*,*P*_(1,1)_,*P*_(1,−1)_) of the order parameter is given by the Boltzmann factor ∼exp[−F(*φ*,*P*_(1,1)_, *P*_(1,−1)_)/(*k*_B_*T*)], where *F* is the free energy. Finding the position of the maximum of the histogram (which is a point in 3D space (*φ*,*P*_(1,1)_,*P*_(1,−1)_)) is then equivalent to minimize the free energy and identifies the stablest phase. This yields sharper transitions than following the thermal average which, with our accessible system sizes, often is not large enough to resolve closely separated transitions. More details are given in Supplementary Figs 9 and 10, and Supplementary Note 5.

## Additional information

**How to cite this article:** Capati, M. *et al*. Electronic polymers and soft-matter-like broken symmetries in underdoped cuprates. *Nat. Commun.* 6:7691 doi: 10.1038/ncomms8691 (2015).

## Supplementary Material

Supplementary Information Supplementary Figures 1-10, Supplementary Notes 1-5 and Supplementary References
