# Supplementary material for: Electronic polymers and soft-matter-like broken symmetries in underdoped cuprates
Source: Nat Commun. 2015 Jul 6;6:7691. doi: 10.1038/ncomms8691 (PMC4506492; doi:10.1038/ncomms8691)
Supplement: Supplementary Information — Supplementary Figures 1-10, Supplementary Notes 1-5 and Supplementary References [file ncomms8691-s1.pdf]

Supplementary Figures

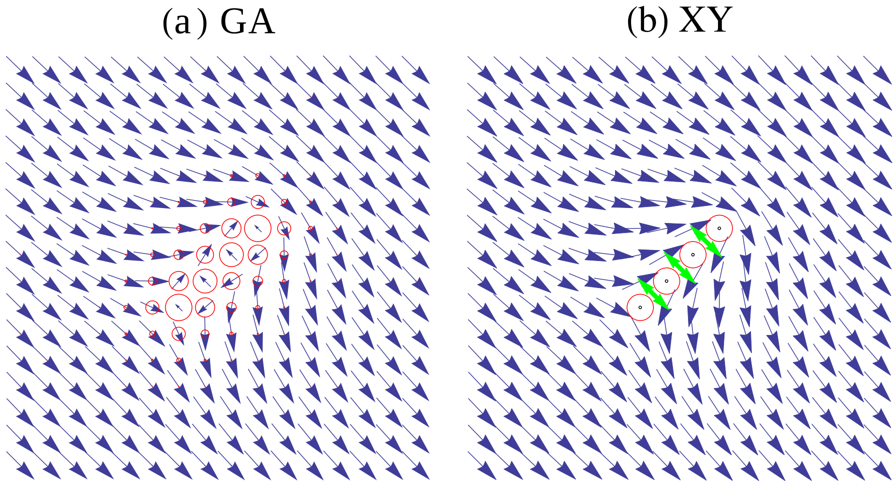

Supplementary Figure 1: **Charge segments and magnetic distortion.** We show a segment containing 4 holes (2 vortex-antivortex pairs) on a 16×16 lattice. The blue arrows are the staggered magnetization (i.e. spins in one sublattice are reversed for better visualization of textures). In (a) we show results in the Gutzwiller approximation for the Hubbard model with $U/t = 8$, $t'/t = -0.2$ and in (b) we show results in the XY-model with $J'/J = 1.2$ (b). In (a) the radius of the circles indicates the (hole) charge density and in (b) corresponds to a vacant site. The green segments in (b) represent the frustrating antiferromagnetic coupling J' across the pairs.

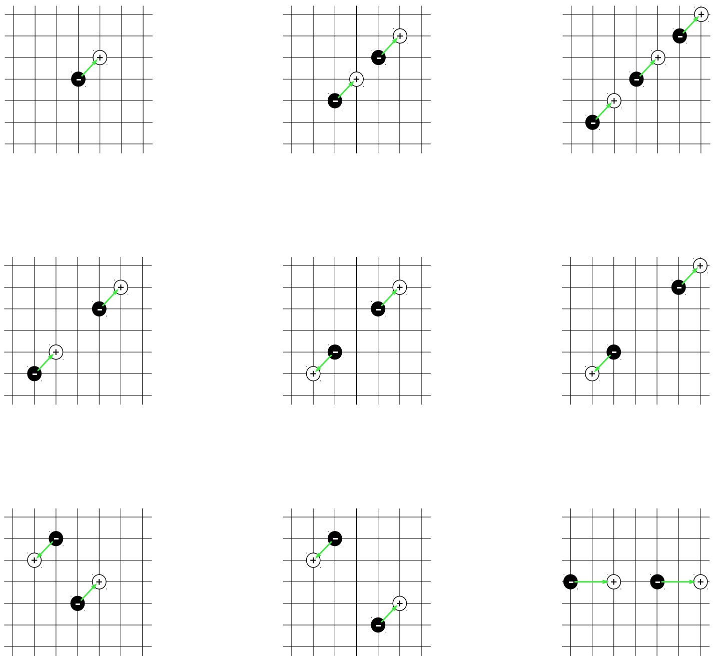

Supplementary Figure 2: **Configurations of topological charges.** We show configurations used to compute the short-range terms in the effective interaction. The black and white circles represent respectively negative and positive charges. The green arrows represent the dipole moments generated by the pairs of opposite charges.

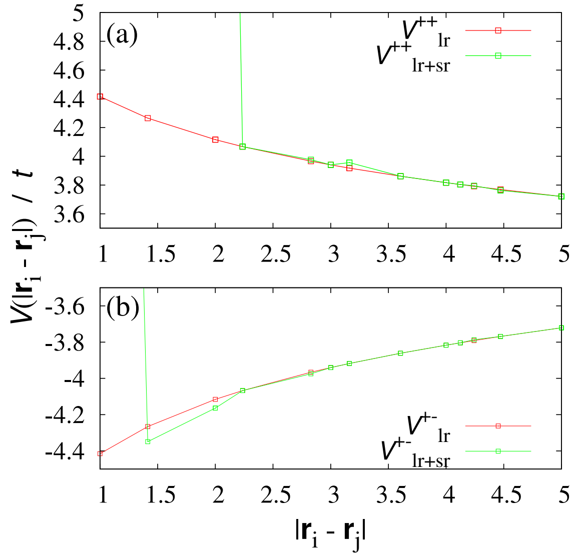

Supplementary Figure 3: **Effective interaction.** Interaction between two topological charges of the same sign (a) and different sign (b) for discrete values of the distance (measured in units of the lattice constant). The long-range part of the potential (red line) is compared to the total potential including both the short- and the long-range part (green line).

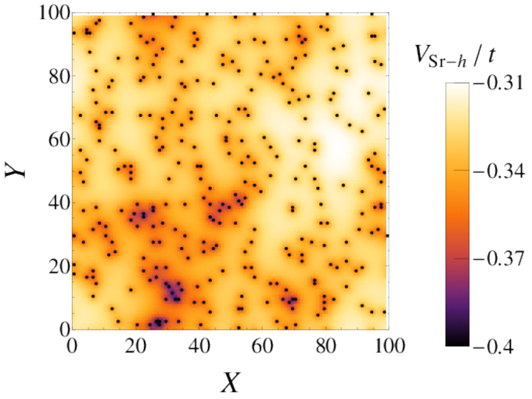

Supplementary Figure 4: **Quenched disorder.** Density plot of the attractive potential generated by the negative Strontium ions on the nearest CuO plane, for $Q_{\mathrm{ion}}/Q_{\mathrm{rep}} = 0.125$. The darker is the color the deeper is the potential well. The projection of the position of the ions on the plane is represented by the black points.

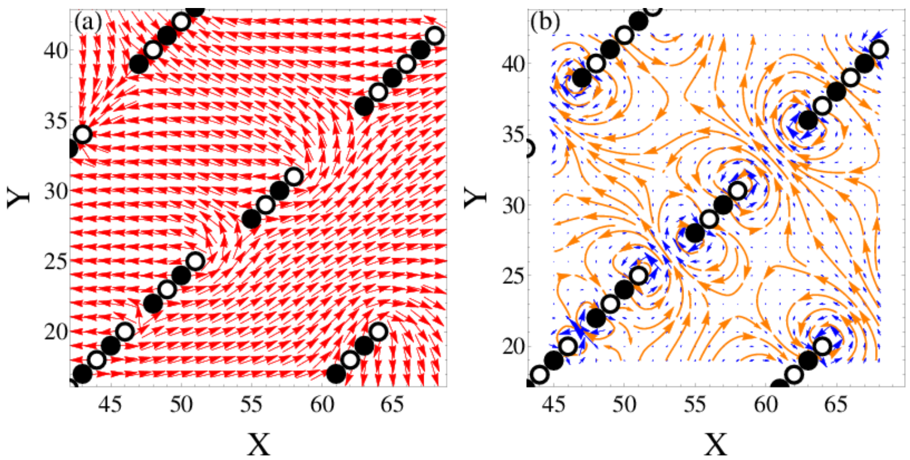

Supplementary Figure 5: **Zoom into segments** (a) Configuration of the selected region for $T = 38\,\mathrm{K}$ in the clean limit: the positive and the negative topological charges are represented respectively by open and closed black circles. The red arrows are the staggered spins in each site. (b) Schematic representation of the spin current: the blue arrow in each site is the average value of the currents in the four bonds connecting the site with its nearest neighbours. The orange streamlines show the general trend of the overall current.

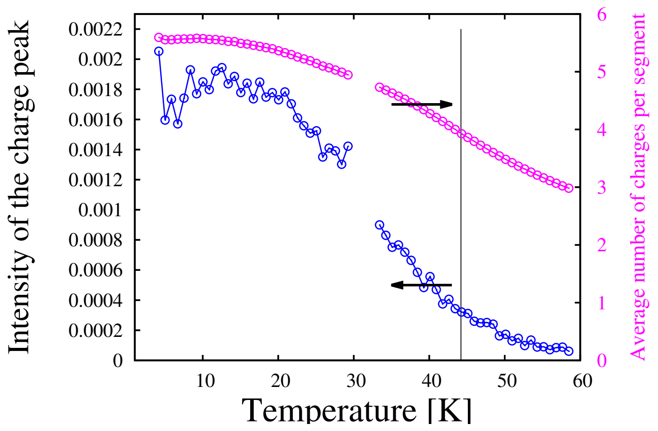

Supplementary Figure 6: **Charge peaks and average segment length.** We show the temperature dependence of the main charge peak (left scale) and the average charges per segment which coincides with the average length divided $\sqrt{2}$ (right scale). The arrows help to identify the scale associated with the data. Disorder is fixed at $Q_{\text{ion}}/Q_{\text{rep}} = 0.125$.

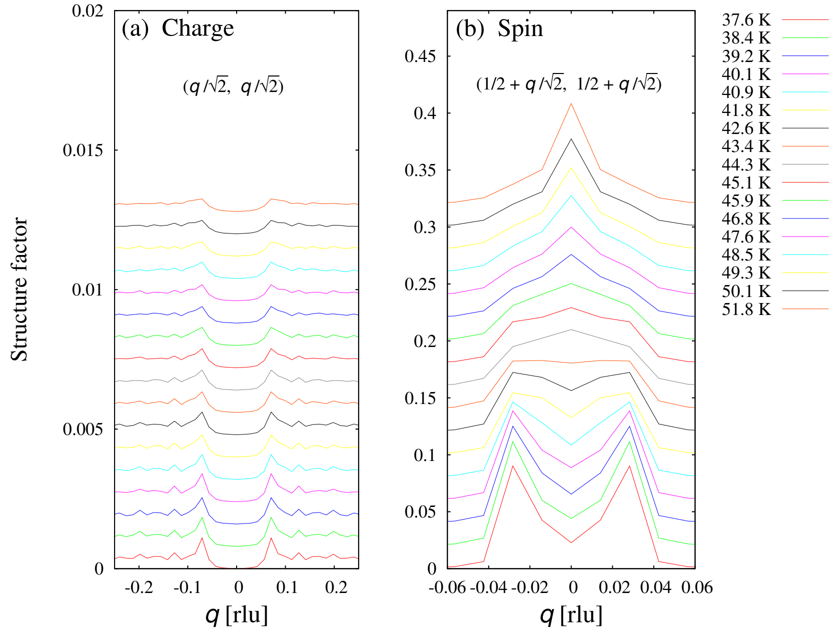

Supplementary Figure 7: **Structure factor for moderate disorder.** Diagonal cut of the (a) charge and (b) spin structure factors for a range of temperatures for $Q_{\text{ion}}/Q_{\text{rep}} = 0.125$.

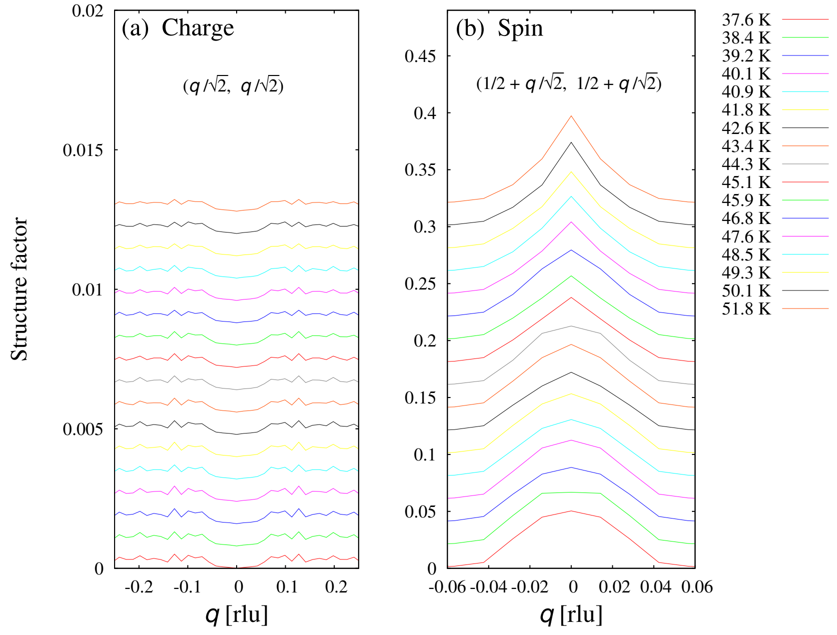

Supplementary Figure 8: **Structure factor for large disorder.** Diagonal cut of the (a) charge and (b) spin structure factors for $Q_{\rm ion}/Q_{\rm rep} = 0.25$ and a range of temperatures. Going up within the panel the temperature increases.

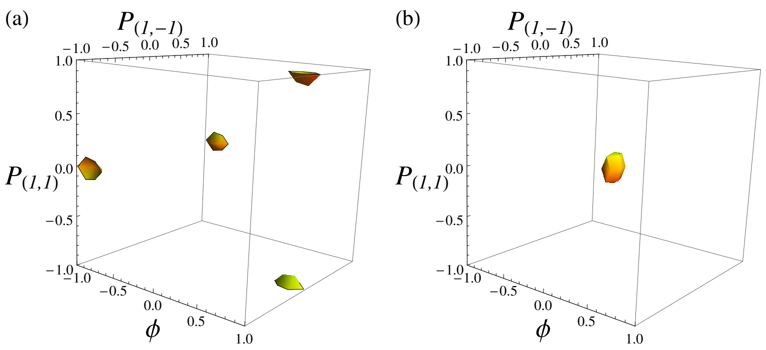

Supplementary Figure 9: **Histogram in 3D order parameter space.** Example of an Monte Carlo simulation of the clean system for (a) $T = 38$ K and (b) $T = 50$ K, respectively. Statistically independent points where binned according to the value of the three order parameters $(\phi, P_{(1,1)}, P_{(1,-1)})$ in 17 bins per each side (totally 4913 cells). An isosurface with 500 points per bin is plotted. Colors help to visualize the three-dimensional form and have no other meaning.

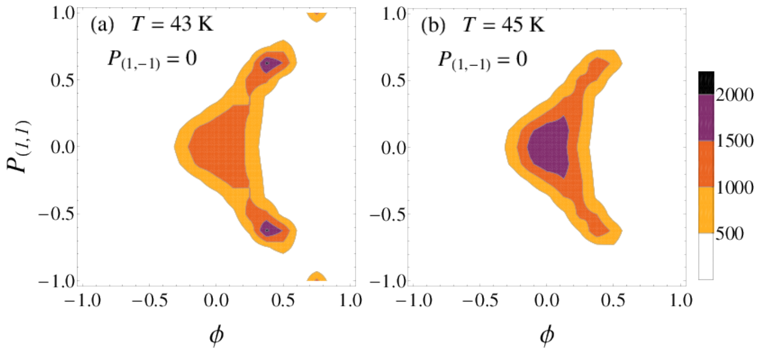

Supplementary Figure 10: **Nature of transition.** Histograms of the order parameters in the 2D space $[\phi, P_{(1,1)}]$ characterized by the plane $P_{(1,-1)} = 0$, for the moderately disordered case $Q_{\text{ion}}/Q_{\text{rep}} = 0.125$, immediately below (a) and above (b) the first-order phase transition between the disorder and the ferronematic state.

Supplementary Notes

Supplementary Note 1: Calculation of the effective interaction among topological charges

Our starting point is the one-band Hubbard model describing the CuO_2 planes, of cuprates

$$\mathcal{H}_{Hub} = -t \sum_{\langle i,j \rangle,\sigma} c^{\dagger}_{i,\sigma} c_{j,\sigma} - t' \sum_{\langle\langle i,j \rangle\rangle,\sigma} c^{\dagger}_{i,\sigma} c_{j,\sigma} + U \sum_{i} n_{i,\uparrow} n_{i,\downarrow}, \tag{1}$$

where $c_{i,\sigma}$ ($c^{\dagger}_{i,\sigma}$) destroys (creates) an electron with spin σ at the site i, and $n_{i,\sigma} = c^{\dagger}_{i,\sigma} c_{i,\sigma}$ is the number operator at the site i. The two summations are restricted over nearest-neighbor sites $\langle i,j \rangle$ and next-nearest neighbor sites $\langle\langle i,j \rangle\rangle$. U is the on-site Hubbard repulsion while t and t' denote the nearest and the next-nearest neighbor hopping, respectively. We measure all distances in units of the lattice constant ($a = 1$). In addition we fix the numerical value of the parameters at $t'/t = -0.2$, $U/t = 8$, with $t = 360$ meV as in Ref. [1]. This parameter set is very close to the one of Refs. [2,3] which has been shown to reproduce the spectrum of magnetic excitations and the charge transfer gap. From the Gutzwiller approximation (GA) energy increment in the presence of a uniform spiral we compute the bare spin stiffness of the model to be $\rho_s = 0.11\, t = 39.6$ meV.

To obtain the effective interaction among topological charges we have performed GA computations of model Eq. (1). We have considered the energy of several metastable configurations in which an even number of holes is self-trapped in vortex-antivortex (VA) pairs located at several position in the lattice, with a one-to-one correspondence between pairs of holes and VA pairs. Supplementary Figure 1(a) shows a typical configuration for a 16×16 cluster with $N_c = 4$ holes forming two VA pairs aggregated in a four-site segment.

Mapping to an effective XY model. Since the textures we considered are long-ranged and the cluster has periodic boundary conditions, the energy of each configuration is strongly dependent on the system size which is limited to small clusters (16×16) in the Hubbard model. One expects, however, that the short-range part of the interaction, which is due to the overlap of the hole-wave functions within the core of the segment, is well converged in our cluster. Fortunately, because the lowest energy textures we find are planar, the long-range part of the interaction can be well reproduced with an effective XY model which allows to extrapolate the results to very large clusters.

In the effective XY model the localized holes are replaced by vacant sites and an effective frustrating interaction is introduced between the spins in sites nearest neighbors to the holes. A similar approach was used in Ref. [4] although in that paper the minimal topological dipole corresponds to one hole instead of two holes as in our case. In fact in our GA computations we find that the one hole state is a collinear spin polaron and only for two holes or more the GA yields the dipolar distortion.

Supplementary Figure 1(b) shows an example with the green segments representing the effective interactions across the chain of VA pairs.

The effective hamiltonian we use reads,

$$\mathcal{H}_{XY} = \sum_{i,j} J_{ij} \left[s_i^x(\mathbf{r}_i) s^x(\mathbf{r}_j) + s_i^y(\mathbf{r}_i) s^y(\mathbf{r}_j) \right], \tag{2}$$

where $J_{ij} = J$ for nearest-neighbor sites if i and j are not vacant sites, $J_{ij} = J' > 0$ across each VA pair (which plays the role of an elementary topological dipole) and $J_{ij} = 0$ elsewhere. J is

adjusted to match the stiffness of the Hubbard model in the GA and J' is adjusted so that the dipole strength of the two models agree as explained below.

In order to obtain the dipole strength p_0 of a dipole oriented along the $(1, 1)$ direction, we compute the average spin current in the direction $\hat{\mathbf{u}} = \frac{1}{\sqrt{2}}(1, -1)$ by averaging over a line parallel to the dipole moment. The spin current can be shown from the mapping to a Coulomb gas model to be directly proportional to the dipole strength,

$$\langle \nabla\theta \cdot \hat{\mathbf{u}} \rangle = \frac{2\pi p_0}{L^2}. \tag{3}$$

Then we adjust J' of the XY model in such a way to match the dipole strength of the Hubbard model computed in the same way. This ensures that the long-range part of the energy of the XY model coincides with the long-range part of the energy of the Hubbard model for the same texture.

Supplementary Figure 2 shows the configurations considered to obtain the effective interaction among the topological charges once the mapping to the Coulomb gas is done.

In order to extrapolate to large clusters we assume that the energy of each configuration for the cluster of size L can be written as the sum of short-range (sr) terms plus long-range (lr) terms,

$$E_{\text{Hub},L\times L} = E_{\text{Hub,sr},L\times L} + E_{\text{lr},L\times L}.$$

By construction $E_{\text{lr},L\times L}$ coincides with the same term in the effective XY model, therefore we can write

$$E_{\text{XY},L\times L} = E_{\text{XY,sr},L\times L} + E_{\text{lr},L\times L}.$$

The energy of the Hubbard model for a large cluster of size L' is obtained assuming that the short-range terms do not depend on the system size according to

$$E_{\text{Hub},L'\times L'} = E_{\text{Hub},L\times L} - E_{\text{XY},L\times L} + E_{\text{XY},L'\times L'}.$$

Next according to the Coulomb gas mapping [5] we assume that the long-range part of the energy can be represented by topological charges of strength $\pm k$.

Examining the GA textures we find that topological charges are centered close to (but not exactly at) lattice sites. Since in the Monte Carlo computation only the dipole strength $p = \pm kl$ plays a role we use the freedom on k and l to force the topological charges to be located exactly at lattice sites. k was determined in such a way that when a dipole $\mathbf{p}$ is formed with charges on the diagonal of a plaquette of length $l = \sqrt{2}a$, the dipole moment $p_0 = \pm kl$ reproduces that of the GA and XY computations yielding $k = 0.8$.

Next, we decompose $E_{\text{Hub},L'\times L'}$ for N_h holes in the different contributions,

$$\begin{aligned} E_{\text{Hub},L'\times L'} &= E_{\text{AF},L'\times L'} + N_h\epsilon_0 \\ &+ \sum_{i<j} \delta V^{\eta_i,\eta_j}(r_{ij}) + 2\pi\rho_s k^2 \sum_{i<j} \eta_i\eta_j \log(r_{ij}). \end{aligned} \quad (4)$$

Here, $E_{AF,L'\times L'}$ is the energy of the antiferromagnetic solution in a cluster of the same size, r_{ij} is the distance between topological charges i and j, $\eta_i = \pm$ is the sign of the topological charges, ϵ_0 is a self-energy term independent of the distance among the topological charges. The last two terms represent the long- and short-range parts of the interaction. Our remaining task is to determine the short-range interaction.

GA computations for the configurations shown in Supplementary Figure 2 allow to equate the corresponding sums $N_h\epsilon_0+\sum_{ij}\delta V_r^{\eta_i,\eta_j}$ to known energies. This provides a system of equations which can be inverted to obtain the various $\delta V_r^{\eta_i,\eta_j}$. Notice that ϵ_0 is irrelevant in the Monte Carlo computations since we work at a fixed number of particles.

Supplementary Figure 3 shows the total potential $V(r)$ between two topological charges of the same sign (a) and of different sign (b), as a function of their distance r, including short- and long-range parts (green lines) and compared with the long-range part alone (red lines).

We have assigned high energy values $(10t)$ to the terms $\delta V_{r=1}^{+-}$ and $\delta V_{r=2}^{++}$, since these configurations cannot be stabilized even as metastable states. This effectively has the effect of eliminating these configurations from the Monte Carlo computations, so that the computations will not depend on the precise value of these constants.

Notice that the effective interaction between topological charges should not be taken literally. Indeed if two holes are separated at a long distance the Coulomb gas energy is logarithmically large because it assumes the vortex and antivortex configuration to persists indefinitely. Instead for large distances the GA solution converges to two collinear spin polarons which have a smaller energy. Thus for those configurations the effective interaction breaks down. The interaction makes sense only if the VA pairs are at short distances and form dipoles. Thus in the Monte Carlo computations we restrict the temperature range in such a way that this condition is met.

Supplementary Note 2: Treatment of quenched disorder due to dopants

In the effective Coulomb gas model for the Monte Carlo simulations, we take into account the disorder due to out-of-plane ions. Since each V or A is associated with a hole carrier, we associate to each V or A also a real positive Coulomb charge. These hole charges feel the disordered potential out of the plane generated by the doping out-of-plane ions.

We use as a model system $La_{2-x}Sr_xCuO_4$ in which the ions are positive Sr^{2+} which replace La^{3+} ions out of the plane. With respect to a uniform background, each Sr ion can be modeled as a point-like negative charge which generates a long-range Coulomb potential. Thus in order to represent approximately the disorder introduced by dopants we place a fraction x of point-like negative charges at a distance $\bar{d} = 0.58a$ from the plane. The charges are located above the center of the in-plane plaquettes formed by 4 sites and the plaquettes are chosen randomly. For each value of the disorder strength $Q_{\rm ion}/Q_{\rm rep}$ in the computation of the phase diagram on the main article, we take four random distributions of the point-like negative charges, and the Monte Carlo results are averaged over these four configurations.

Supplementary Figure 4 shows the potential field generated by a given configuration of disorder in the plane where the holes move,

$$V_{\rm ion}(x,y) = -\sum_i \frac{Q_{\rm ion}}{|\mathbf{r}-\mathbf{R}_i|}$$

where $\mathbf{R}_i$ is a three dimensional vector representing the position of the i-th impurity and the sum runs over all impurities. $\mathbf{r} = (x, y, 0)$ is the three dimensional coordinate of the two-dimensional

lattice site (x, y).

Supplementary Note 3: Detailed view of the segments and the correspondent spin configuration in the ferrosmectic phase

In Supplementary Figure 5 we report the zoom of a snapshot of our lattice in the Monte Carlo simulation of the clean system in the ferrosmectic phase ($T = 38\,\mathrm{K}$). In particular the two panels show (a) the topological charge and staggered spin configuration, and (b) the spin current, calculated treating the topological charges as spin vortices and antivortices within the XY model.

These panels show how the charge and spin channels are strictly connected. The first picture shows a local inversion symmetry breaking due to the polarized segments. Furthermore, looking at the spin texture resulting from the disposition of the topological charges, it can be noticed that each segment generates an anti-phase domain, i.e. a jump of nearly π in the phase of the spins.

Panel (b) is a representation of the spin current. Although the latter is a quantity defined in each bond as $I_{ij} = J_{ij}(\mathbf{s}_i \times \mathbf{s}_j) \cdot \hat{z}$, it is represented in each site as the average value of the currents in the four bonds connecting the site with its nearest-neighbor sites. Since the spin current is a conserved quantity, we can define a vector field, which can be easily visualized by means of the flux lines. They are represented as orange curved arrows in Supplementary Figure 5(b), and show manifestly the inversion symmetry breaking in the spin channel. Notice that the spin current is closely related to the vector spin chirality defined in the Methods section of the main article.

Supplementary Note 4: Temperature and disorder dependence of the structure factors

Here we show additional plots of the charge and spin structure factors which allow us to estimate the strength of disorder in the model compared with experiment.

Supplementary Figure 6 shows that the charge peaks do not show any anomaly as function of temperature at the temperature of the transition. The same is true for the average length of the segments.

Supplementary Figure 7 shows the temperature dependence of the spin and charge structure factors for small disorder ($Q_{\rm ion}/Q_{\rm rep} = 0.125$), while Supplementary Figure 8 shows the same quantities for large disorder ($Q_{\rm ion}/Q_{\rm rep} = 0.25$). We see that in the case of large disorder incommensurate spin structures are not resolved down to the lowest temperatures. Since in the experiments these peaks are broad but well resolved at low temperatures (see Refs. [7–10]) we conclude that the realistic value of $Q_{\rm ion}/Q_{\rm rep} < 0.25$. For completeness Supplementary Figure 8 (a) shows that charge ordering peaks are also absent.

Figure 7(b) show that for $Q_{\rm ion}/Q_{\rm rep} = 0.125$, the low temperature width of the peaks in the spin structure factor is very similar to the experimental structure factor (see Refs. [9–11]). It also show that, as a function of temperature, the structure factor evolves from incommensurate peaks at low temperatures to a commensurate peak above the ferronematic ordering temperature reported in the main article (45 K). The same data are plotted convoluted with a Gaussian in panel (a) of Figure 3 of the main article.

Supplementary Note 5: Nature of phase transitions

The study of phase transitions with Monte Carlo calculations on finite size clusters poses obvious problems. In particular it is of physical relevance to distinguish whether a vanishing order parameter is small because the symmetry of the phase is not broken or because the Monte Carlo evolution is "ergodically" exploring and spending equal times in regions with opposite values of the order parameters. To this purpose, to find the stablest phases at a given temperature we use the method explained in a previous work [6], which monitors and records the Monte Carlo evolution of the order parameter(s). In our present case, we characterize Monte Carlo generated configurations by the instantaneous value of a three-dimensional (3D) order parameter defined as the vector $(\phi, P_{(1,1)}, P_{(1,-1)})$.

Each different phase is characterized by a "cloud" of points in different regions of the 3D space. In order to characterize these different phases, we compute the 3D density distribution of points at each temperature of the simulation. The points are expected to be distributed with a Boltzmann weight $\exp\left[-F(\phi, P_{(1,1)}, P_{(1,-1)})/(k_B T)\right]$, so that higher density of points corresponds to the lower free energy F and indicates the stablest phase. This distribution can be visualized by plotting isosurfaces with a given number of points per unit volume. As an example we report in Supplementary Figure 9 this type of analysis for a low temperature state and an high temperature state, in a clean system.

Since at low temperature (Supplementary Figure 9(a)) the highest density of points occurs inside the two regions around $\phi \sim +1$ and $P_{(1,1)} \sim \pm 1$, and the two regions around $\phi \sim -1$, and

$P_{(1,-1)} \sim \pm 1$, this phase is identified as ferrosmectic or ferronematic depending on the presence of charge ordering, which can be checked *a posteriori* from the charge structure factor. At high temperature [Supplementary Figure 9(b)] the densest cloud of points occurs in correspondence of the zero value of the three order parameters indicating that at this temperature the system is in the disordered phase.

The phase transition can be visualized more easily by plotting 2D cuts of the 3D histogram, i.e. we plot only the plane $P_{(1,-1)} = \mathrm{constant}$, for $\phi > 0$, or $P_{(1,-1)} = \mathrm{constant}$, for $\phi < 0$. The constant is determined in such a way that the plane contains the bin with the maximum number of points of all the 3D histogram which usually yields a value for the constant nearly zero. So, if $\phi > 0$, we take the plane $P_{(1,-1)} = 0$ and plot the 3D bins in this plane as a 2D histogram, whose axes are ϕ and $P_{(1,1)}$. In the case of Supplementary Figure 9(a) this yields a 2D histogram with maxima at $\phi \sim 1$ and $P_{(1,1)} \sim \pm 1$ while in the disordered case a single maximum appears at the origin (2D histograms not shown).

Using this procedure Supplementary Figure 10 shows that the phase transition from the disordered phase to the ferronematic phase remains sharp in the presence of realistic disorder. Indeed, in a narrow temperature range the maximum of the distribution shifts from finite values of polarization and nematicity (a) to the disordered phase characterized by a maximum of the distribution close to the origin (b).

Supplementary References

1. Seibold, G., Capati, M., Di Castro, C., Grilli, M. & Lorenzana, J. Hidden ferronematic order in underdoped cuprates. *Phys. Rev. B* **87**, 035138 (2013).

2. Lorenzana, J., Seibold, G. & Coldea, R. Sum rules and missing spectral weight in magnetic neutron scattering in the cuprates. *Phys. Rev. B* **72**, 224511 (2005).

3. Lorenzana, J. & Seibold, G. Unified description of charge and spin excitations of stripes in cuprates. *Physica C* **460-462**, 271–274 (2007).

4. Aharony, A., Birgeneau, R., Coniglio, A., Kastner, M. & Stanley, H. Magnetic phase diagram and magnetic pairing in doped La_2CuO_4. *Phys. Rev. Lett.* **60**, 1330–1333 (1988).

5. Minnhagen, P. The two-dimensional coulomb gas, vortex unbinding, and superfluid-superconducting films. *Rev. Mod. Phys.* **59**, 1001–1066 (1987).

6. Capati, M., Grilli, M. & Lorenzana, J. Nematic phase without Heisenberg physics in FeAs planes. *Phys. Rev. B* **84**, 214520 (2011).

7. Cheong, S. W. *et al.* Incommensurate magnetic fluctuations in $La_{2x}Sr_xCuO_4$. *Phys. Rev. Lett.* **67**, 1791–1794 (1991).

8. Yamada, K. *et al.* Doping dependence of the spatially modulated dynamical spin correlations and the superconducting-transition temperature in $La_{2-x}Sr_xCuO_4$. *Phys. Rev. B* **57**, 6165–6172 (1998).

9. Wakimoto, S. *et al.* Observation of incommensurate magnetic correlations at the lower critical concentration for superconductivity in $La_{2-x}Sr_xCuO_4$ ($x = 0.05$). *Phys. Rev. B* **60**, R769–R772 (1999).

10. Wakimoto, S. *et al.* Direct observation of a one-dimensional static spin modulation in insulating $La_{1.95}Sr_{0.05}CuO_4$. *Phys. Rev. B* **61**, 3699–3706 (2000).

11. Drachuck, G. *et al.* Comprehensive study of the spin-charge interplay in antiferromagnetic $La_{2-x}Sr_xCuO_4$. *Nat. Commun.* **5**, 3390 (2014).
